# Exploring life skills, research adaptation, and team building among basic medical postgraduates

**DOI:** 10.3389/fmed.2025.1638333

**Published:** 2025-09-17

**Authors:** Qiqi Zhang, Xiaoqing Yuan, Ziyu Liu, Jie Liu, Diabate Ousmane, Junpu Wang

**Affiliations:** ^1^Department of Pathology, Xiangya School of Basic Medical Sciences, Central South University, Changsha, China; ^2^Department of Pathology, Xiangya Hospital, Central South University, Changsha, China; ^3^Medical Research Center, Sun Yat-Sen Memorial Hospital, Sun Yat-Sen University, Guangzhou, China; ^4^Guangdong Provincial Key Laboratory of Malignant Tumor Epigenetics and Gene Regulation, Guangdong-Hong Kong Joint Laboratory for RNA Medicine, Sun Yat-Sen Memorial Hospital, Sun Yat-Sen University, Guangzhou, China; ^5^Department of Pathology, Ultrapathology (Biomedical Electron Microscopy) Center, Xiangya Hospital, Central South University, Changsha, China; ^6^FuRong Laboratory, Changsha, China; ^7^National Clinical Research Center for Geriatric Disorders, Xiangya Hospital, Central South University, Changsha, China

**Keywords:** basic excellence 2.0, graduate student, educational institutions, scientific research, supervisor

## Abstract

**Introduction:**

Under the background of “basic top-notch 2.0” plan, taking the graduate students of Xiangya School of Basic Medical Sciences, Central South University as an example, to explore their current situations in life, academics, scientific research, and team collaboration, thereby offering insights for enhancing the quality of basic medical graduate education in China, achieving the plan’s objectives, and shaping relevant policies.

**Methods:**

An online anonymous questionnaire was sent to graduate students of basic medicine majors in different grades of Xiangya School of Basic Medical Sciences, Central South University. The questionnaire included 24 questions covering different aspects of graduate life, and the data of 350 valid questionnaires were analyzed. The study reveals diverse academic backgrounds among basic medical graduate students, with nearly half (50%) originating from different disciplines.

**Results:**

Over 14% of students reported encountering learning difficulties, and many tended to seek help from peers rather than supervisors due to limited communication with their advisors. Approximately 80% of graduate students had flexible time management, yet only 26% received sufficient stipends to cover daily expenses, with an equal proportion facing inadequate research funding. Academic engagement was moderate (54.7%), though some students demonstrated low participation enthusiasm. Notably, nearly one-third (33.3%) had no prior research experience before enrollment. While overall satisfaction with supervisors’ academic and research guidance was high (96.29%), senior students exhibited a slight decline in satisfaction. A strong preference for the teamwork environment was reported (86.57%), though the satisfaction of the graduating students with the team working atmosphere was somewhat lower. Finally, 20.86% expressed dissatisfaction with their overall graduate school experience.

**Discussion and conclusion:**

Data analysis revealed pertinent issues, serving as a reference for educational institutions across all levels in formulating policies, such as enhancing graduate student adaptation and academic experience, guiding graduate students to adapt to their specialties, establishing effective communication channels, reasonably managing their own time, boosting funding, constructing academic platforms, facilitating early scientific research collaborations, enhancing guidance, and fostering team building, among others, in order to improve the training model and mechanism and cultivate outstanding medical innovative talents.

## 1 Introduction

Postgraduate medical education in China is divided into two categories: “basic medicine” and “clinical medicine.” Among them, the core mission of basic medical postgraduate education is to cultivate senior scientific research talents, and graduates mainly work in universities, scientific research institutes and biomedical enterprises. The Party and state prioritize nurturing talent in fundamental disciplines. In response to Qian Xuesen’s inquiry, a collaborative effort between the Ministry of Education, the Central Organization Department, and the Ministry of Finance initiated the “Pilot Program for Cultivating Outstanding Students in Fundamental Disciplines” in 2009. This program aims to develop local academic leaders and fortify the groundwork of fundamental disciplines (1–3). Without a solid foundation, the earth will shake and the mountains will collapse—a truth most vividly reflected in the realm of basic science (4). To address bottlenecks in basic science, the Ministry of Education and six other departments launched the upgraded Top-notch Plan 2. in 2018, building upon the previous Top-notch Plan 1 (5). Central South University was designated as the “Training Hub for Exceptional Students in Fundamental Medicine (6).” The training of fundamental medical graduates plays a pivotal role as primary contributors to the success of this initiative. In 2024, the General Office of the Central Committee of the Communist Party of China and The General Office of the State Council issued the “Opinions on Accelerating the High-Quality Development of Doctoral Education,” proposing to optimize the collaborative mechanism and enhance the ability to cultivate top-notch innovative talents (7). Compared with professional postgraduate students in clinical medicine, postgraduate students basic medical graduate students (mostly in academic programs) have diverse disciplinary backgrounds (8). Basic medical research is the cornerstone of medical development. Highly qualified researchers can reveal the mechanisms of disease through in-depth experimental studies and theoretical exploration, providing theoretical support and new treatment methods for clinical medicine. However, numerous challenges also exist. Graduate students often face mental health issues, difficulties adapting to research, challenges in maintaining work-life balance, and unclear academic expectations (9). These problems are particularly pronounced in medical and biomedical disciplines, where the training period is long, stress levels are high, and there are greater demands for publishing results and developing interdisciplinary competencies. Xiangya School of Basic Medical Sciences, Central South University, is a leading institution in medical education in China. The school consistently ranks high in national evaluations of medical education. Xiangya School of Basic Medicine Sciences has a reputation for excellent medical education, comprehensive and rigorous training programs, and active participation in international exchanges and cooperation (10). Committed to high-quality medical education and research, the school provides a representative and significant reference for studying the challenges and experiences of graduate students in medical disciplines in the country. Through a questionnaire-based survey, this study provides an in-depth analysis of the living conditions, research adaptation, and team dynamics among graduate students at Xiangya School of Basic Medical Sciences, Central South University. The findings aim to offer critical empirical evidence to improve graduate training programs and inform education policy formulation in basic medical sciences. This will advance China’s graduate education in this field to new heights and has the potential to enhance the overall quality and effectiveness of postgraduate training in the medical education sector in China.

## 2 Methods

### 2.1 Research design

**Figure 1:**
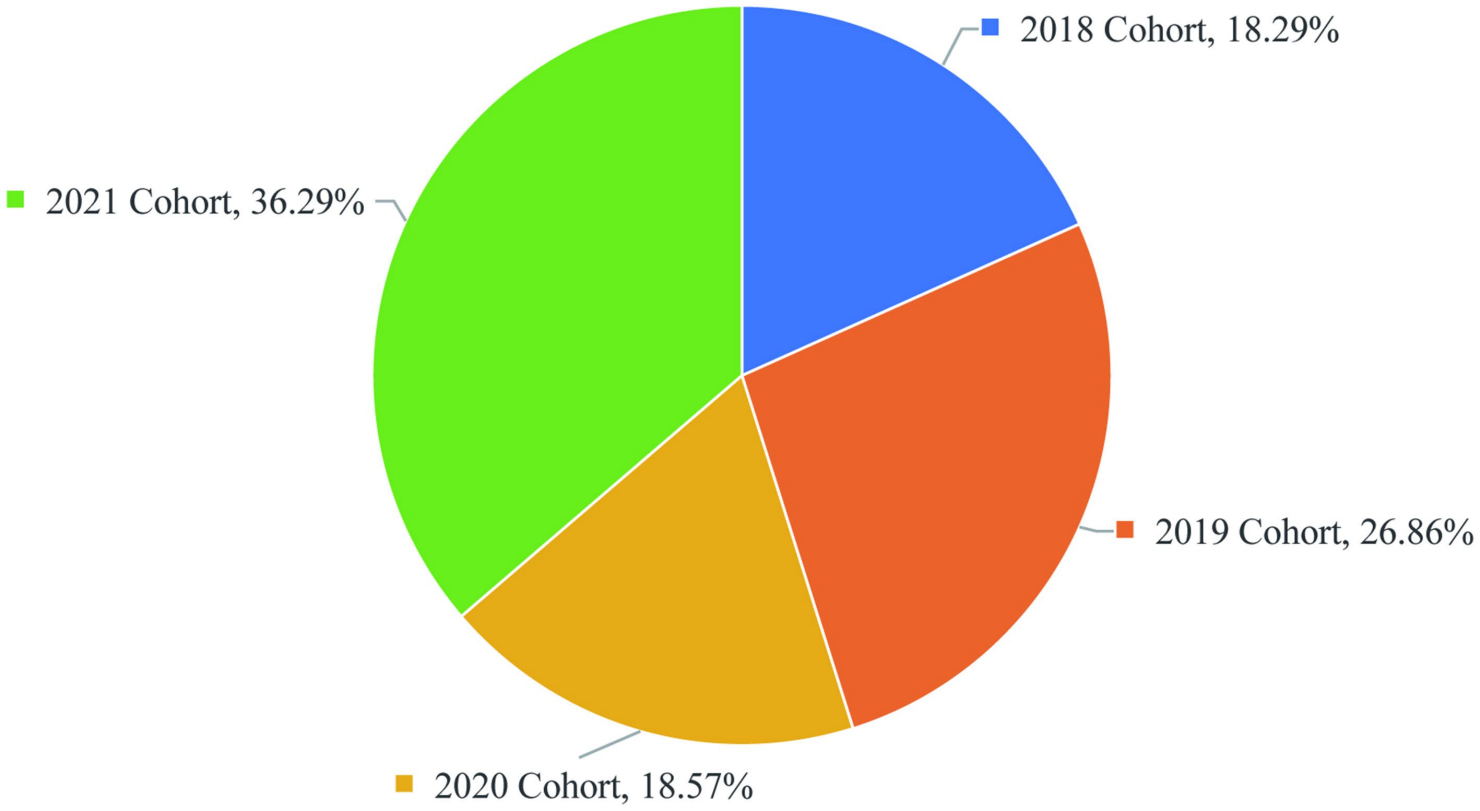
Grades distribution of graduate students.

**Figure 2:**
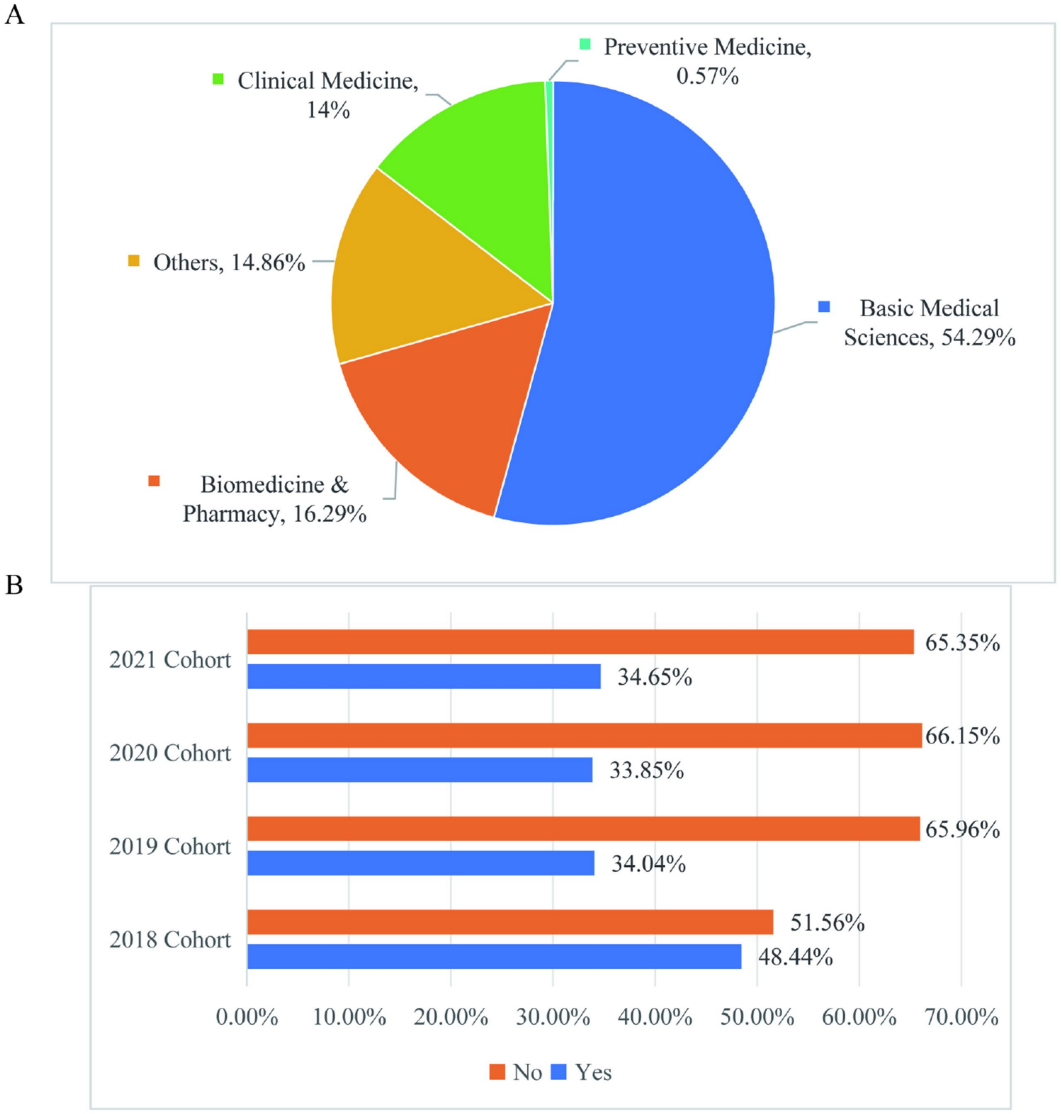
The school structure is diverse. **(A)** Distribution of undergraduate majors in basic medical research. **(B)** The proportion of medical graduate students from different grades who have crossed majors.

This cross-sectional study employed a quantitative survey approach to examine the academic experiences of graduate students at Xiangya School of Basic Medicine during the 2018–2021 academic cohorts. This study focused on graduate students from the 2018–2021 cohorts at Xiangya School of Basic Medical Sciences, Central South University. The cohort distribution was as follows: 18.29% (2018), 26.86% (2019), 18.57% (2020), and 36.29% (2021) (Figure 1). In our study, the cohorts are categorized based on the year of entry into the graduate program. Specifically, the 2018 cohort represents the senior students, while the 2021 cohort represents the junior students. The sample comprised 67.43% female and 32.57% male students. In terms of academic background, 63.14% were non-cross-disciplinary candidates while 36.86% had transitioned from other fields. The survey instrument included 24 questions employing both single-choice and multiple-choice formats as well as open-ended questions, comprehensively assessing: (1) daily life circumstances, (2) academic workload, (3) research activities, and (4) mentor-mentee relationships within research teams. A total of 350 valid questionnaires were collected for analysis. Only 54.29% of graduate students had majored in basic medical sciences during their undergraduate studies, and most of the other undergraduate students come from clinical medicine, biomedicine and pharmacy, preventive medicine and other majors (Figure 2A). The proportion of cross-majors is similar across grades (Figure 2B).

### 2.2 Research setting

This study was conducted at the Xiangya School of Basic Medical Sciences, Central South University, which has been designated a National “Top-Tier Undergraduate Training Base in Basic Medical Sciences” by the Chinese Ministry of Education. The school offers research-intensive graduate programs characterized by laboratory-based training and close supervisor–mentee collaboration in the basic medical sciences.

### 2.3 Participants and sampling

Population: All enrolled graduate students from the 2018, 2019, 2020, and 2021 cohorts.

Sample size achieved: 350 valid questionnaires.

Sampling method: Use WeChat groups to conduct convenient sampling in the form of questionnaire stars.

Inclusion criteria: (1) registered student at the School of Basic Medical Sciences, (2) enrolled between 2018 and 2021.

Exclusion criteria: students on temporary leave or exchange abroad at the time of the survey.

### 2.4 Data collection procedure

The study utilized a 24-item researcher-developed questionnaire containing both single-choice and multiple-choice formats as well as open-ended questions (Table 1). Although the questionnaire was not formally validated, its items were developed through expert consultation with faculty members. Data were collected via an anonymous online survey distributed through Wenjuanxing (a Chinese platform similar to Qualtrics). Each participant took approximately 5–8 min to complete the survey. Prior to participation, respondents were provided with a detailed explanation of the study’s purpose, and their consent was obtained voluntarily.

**Table 1:** Questionnaire questions and option descriptions.

Question number	Question	Options
Q1	What is your current grade level? (Single-Choice)	2021 cohort 2019 cohort 2020 cohort 2018 cohort
Q2	What is your gender? (Single-Choice)	Female Male
Q3	What type of undergraduate degree do you hold? (Single-Choice)	Basic Medical Sciences Biomedicine & Pharmacy Others Clinical Medicine Preventive Medicine
Q4	Did you take the postgraduate entrance examination across a different major? (Single-Choice)	No Yes
Q5	How do you feel about the current graduate study content? (Single-Choice)	(Non-cross-disciplinary) perceived moderate difficulty (Cross-disciplinary) perceived moderate difficulty (Non-cross-disciplinary) Experienced significant challenges due to large disciplinary gap (Cross-disciplinary) Found relatively easy (Cross-disciplinary) Experienced significant challenges due to large disciplinary gap (Non-cross-disciplinary) Found relatively easy
Q6	When encountering difficulties in study or life, who is the first person you would seek help from? (Single-Choice)	Friends Supervisor Family Research Office Counselor Clinical Instructor
Q7	How would you describe your communication with your supervisor? (Single-Choice)	Once a week Every 2–3 days Twice a week Daily Once a month Twice a month Rarely
Q8	If you rarely communicate with your supervisor, what are the reasons? (Multiple-Choice)	Supervisor’s heavy workload Fear of communicating with supervisor Introverted personality with poor communication skills Dislike supervisor (Blank) Too busy with clinical rotations
Q9	What were the main topics of your communication with your supervisor? (Multiple-Choice)	Academic research and scholarly guidance Career guidance and future planning Psychological/emotional and personal issues Ideological and moral education Clinical work
Q10	What is your current work status? (Single-Choice)	Flexible scheduling (complete tasks within specific periods) Daily fixed times check-ins
Q11	Is the research funding you currently receive sufficient? (Single-Choice)	No Yes
Q12	Can the monthly allowance you receive fully cover your daily expenses? (Single-Choice)	Can be covered Cannot be covered
Q13	During the training period, has your supervisor arranged for you to attend any international or domestic academic conferences? (Single-Choice)	No Yes
Q14	Have you ever participated in your supervisor’s research projects or other scientific programs? (Single-Choice)	No Yes
Q15	Have you actively participated in the Graduate Academic Activity Month or the Graduate Innovation Research Support Program application? (Single-Choice)	No Yes
Q16	If you do not participate in the academic activities of the college or apply for graduate research projects, what are the reasons? (Multiple-Choice)	Lack of adequate research skills Do not know Ignore relevant notifications Not interested Too busy with clinical work
Q17	Before your enrollment, had you been exposed to research work? (Single-Choice)	No Yes
Q18	Are you satisfied with your supervisor’s research and academic guidance? (Single-Choice)	Very satisfied Satisfied Moderately satisfied Dissatisfied
Q19	Do you like the working atmosphere of your current research group? (Single-Choice)	No Yes
Q20	How satisfied are you with your current graduate life? (Multiple-Choice)	Satisfied Very satisfied Somewhat dissatisfied Completely dissatisfied
Q21	In your opinion, what problems do you currently have? (Multiple-Choice)	Other Lack of motivation to learn; weak research skills Ideological or psychological issues; poor stress tolerance Ethical and academic integrity issues
Q22	Have you received help from classmates in your study or daily life? (Single-Choice)	No Yes
Q23	In response to the problems you have identified in yourself, what do you plan to do? (Open-ended)	–
Q24	What suggestions or comments do you have regarding postgraduate program management? (Open-ended)	–

### 2.5 Data analysis strategy

Statistical analyses were conducted using SPSS 18.0. Descriptive statistics (means ± standard deviations) summarized continuous variables. Group comparisons employed independent-samples *t*-tests, with statistical significance defined as *p* < 0.05 for all inferential analyses.

### 2.6 Ethical considerations

The study maintains confidentiality through anonymized data collection and secure server storage. Participants are informed that they have the right to withdraw without penalty.

### 2.7 Trustworthiness of the study

The study protocol was reviewed and approved by the Institutional Review Board of the School of Basic Medical Sciences, Central South University (Approval No. IRB-2023-KT137). Four measures were taken to enhance the trustworthiness of the data:

- Credibility—all questionnaire items were pilot-tested with target users and refined before formal deployment.
- Dependability—the survey platform automatically generated time-stamped response logs and used IP-address checks to prevent duplicate entries.
- Confirmability—the storage of raw data is only accessible to the research team.
- Transferability—detailed demographic distributions (cohort, gender, disciplinary background) are reported so that readers can judge the applicability of the findings to other contexts.

### 2.8 Limitations of the methodology

The lack of Likert-scale items constrained nuanced attitude measurement. Self-report bias may affect accuracy of responses regarding sensitive topics like supervisor relationships.

## 3 Results

### 3.1 Learning content difficulty perception

**Figure 3:**
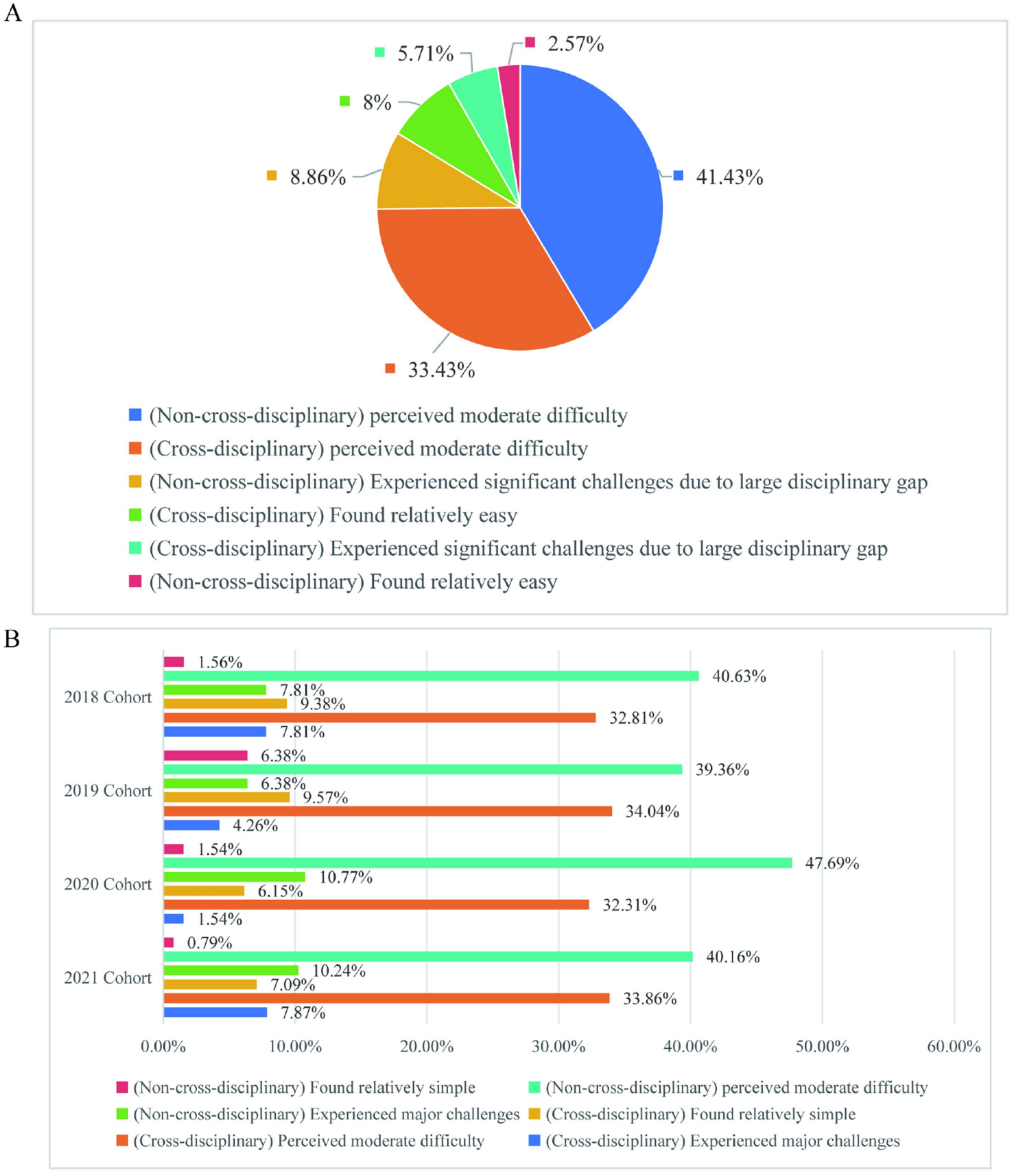
Learning difficulty perception. **(A)** Perception of the subjective difficulty level of the learning content among graduate students in the School of Basic Medicine. **(B)** Perception of subjective difficulty level of learning content among graduate students in the School of Basic Medicine at different grades.

In this study, “learning difficulty” refers to challenges in mastering graduate-level academic content (e.g., theoretical knowledge, technical skills) due to gaps in prior training or inadequate pedagogical support, rather than cognitive dysfunction (which falls under clinical learning disabilities). Figure 3 shows that more than 14% of the students think that there is some difficulty in their current study, while only about 10% feel relatively simple (Figure 3A). It can be seen that the proportion of students who find difficulty in learning is relatively high. At the graduate stage, knowledge comes from many sources: (1) subject teaching; (2) active literature and academic reports; (3) practice. Learning is no longer like undergraduate stage, where students passively accept knowledge, but rather actively organize and collect knowledge. However, for students with relatively weak foundation, reading literature is a big problem. Many students cannot understand the literature thoroughly and master the essence of the literature, so they need to actively practice more. Graduate students of different grades in basic medical schools have similar subjective perception of difficulty in learning content (Figure 3B). This finding indicates that the difficulty in learning content is a common issue among graduate students across different cohorts. It is possible that the current teaching methods and curriculum system may not fully meet the needs of students in terms of helping them adapt to the changes in learning style and content. Therefore, there is still room for improvement in the current teaching of educational institutions at all levels. Optimizing the curriculum system and strengthening learning guidance could be of great significance to help students better adapt to the learning changes at the graduate stage.

### 3.2 Current situation and problems of graduate student and supervisor exchange

**Figure 4:**
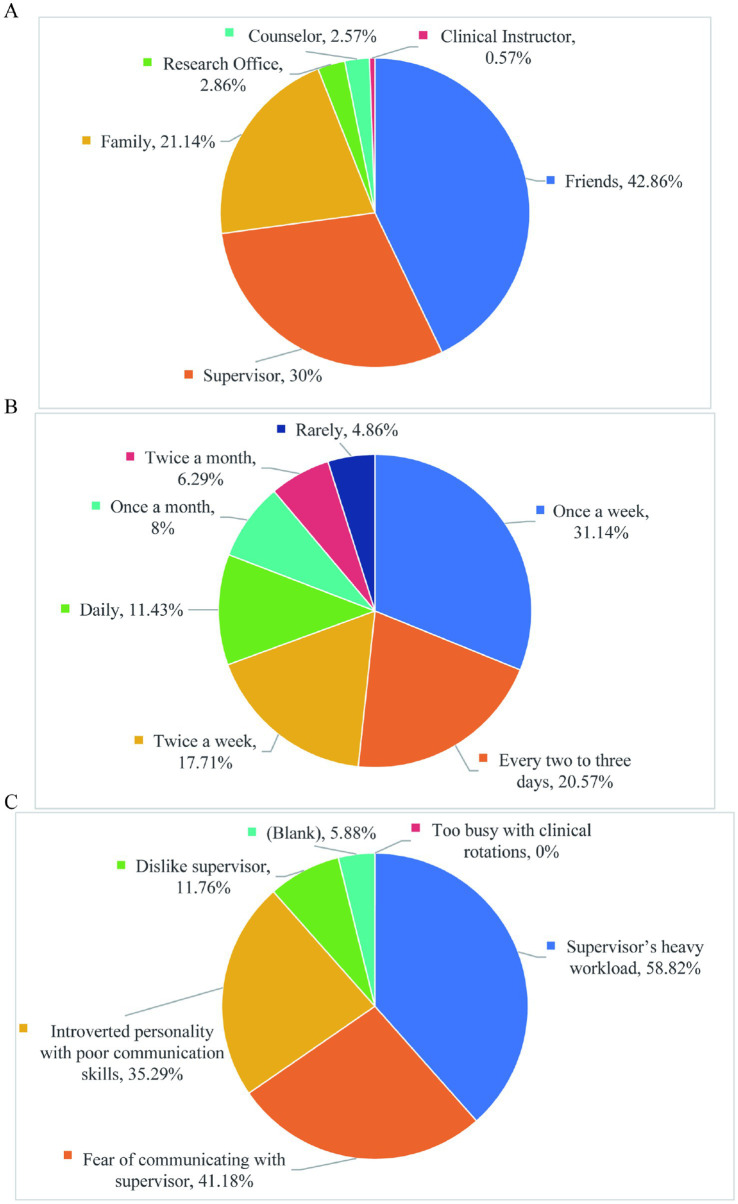
Current situation and problems of graduate student and supervisor exchange. **(A)** When encountering difficulties in studies or life, graduate students should seek help immediately. **(B)** Communication between graduate students of basic medicine and their supervisors. **(C)** Distribution of reasons for limited communication between graduate students and their supervisors.

From the survey results, it can be seen that only 30% of graduate students think of seeking help from their supervisors when they encounter difficulties in their studies or life. Most students will ask their friends for help, and a few students will ask their families for help (Figure 4A). In terms of the frequency of communication with supervisors, a few students only communicate with supervisors once a month (Figure 4B). The main reason for less communication with supervisors is that supervisors are busy (Figure 4C). However, it is important to note that there could be multiple factors influencing students’ preferences for seeking help and communication frequency, such as power dynamics, self-directed learning tendencies, and other contextual factors. This indicates that the teacher-student communication mechanisms in China’s educational institutions still require improvement. Establishing efficient communication is crucial for enhancing postgraduate training quality, as it directly impacts academic guidance effectiveness and students’ psychological wellbeing. Future research could explore these factors in more depth to provide a more comprehensive understanding of the underlying issues.

### 3.3 Time management model

**Figure 5:**
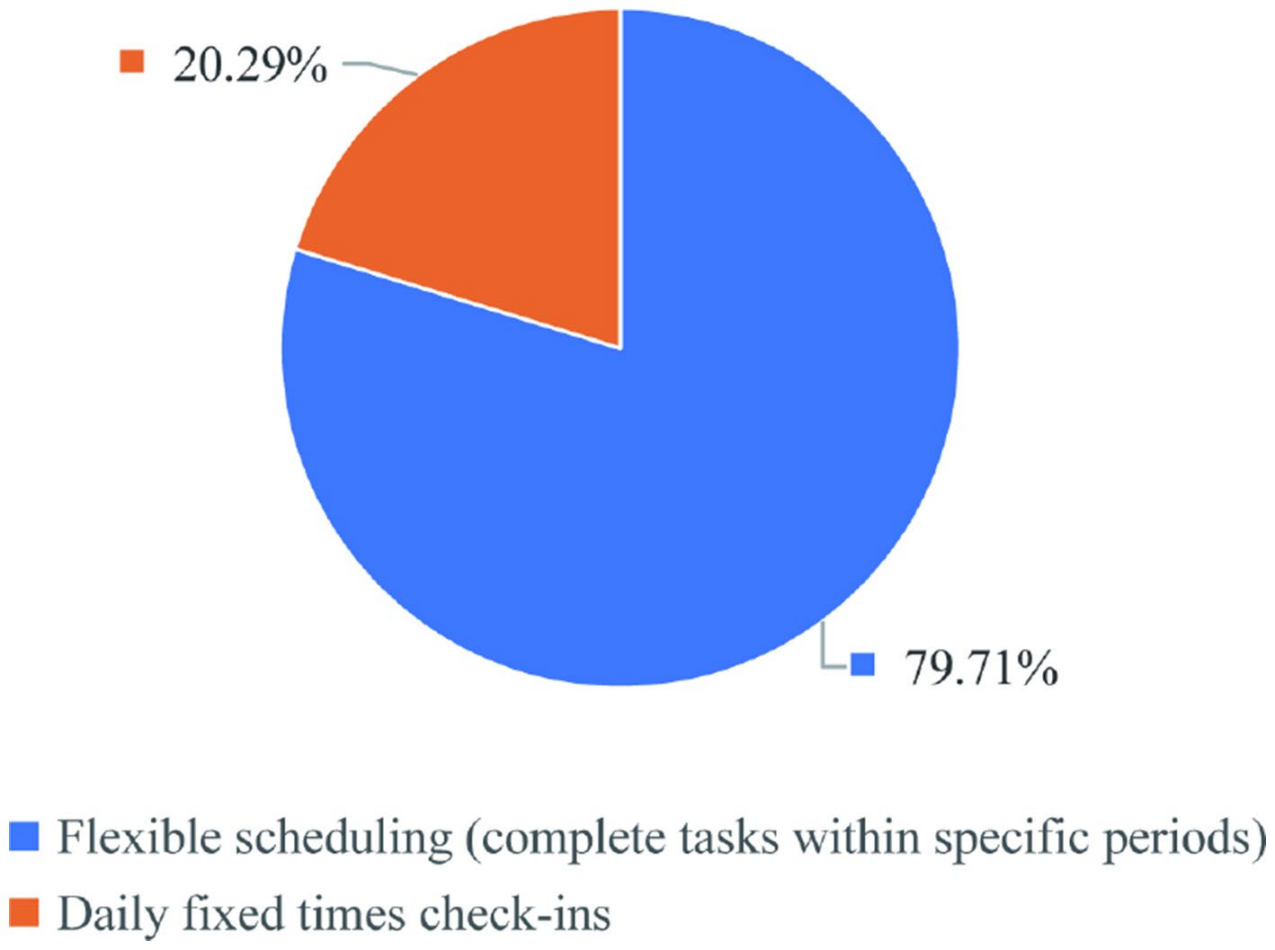
Graduate student schedule.

According to the survey results, about 80% of graduate students have relatively free time schedules, which can be described flexible scheduling, allowing them to complete tasks within defined time periods. This flexible scheduling is particularly conducive to self-directed learning (SDL), a learning approach that has been widely recognized in medical education for its effectiveness in promoting student engagement and efficiency. About 20% of graduate students have relatively tight time schedules, which requires daily fixed-time check-ins (Figure 5). It can be seen that most graduate students have relatively free time schedules. Flexible time schedules enable students to better manage their learning processes, which is essential for SDL. Graduate students can utilize their free time to access research modules and engage in SDL activities. This not only helps them develop critical thinking skills but also prepares them for future professional practice. So how to arrange time reasonably and grasp the balance between study and life is very important. Scientific research requires a lot of time, but scientific research also requires efficiency. Relevant domestic educational institutions shall carry out relevant guidance to guide students to balance scientific research and life, improve time utilization efficiency, form good time management habits, and have an important impact on ensuring the quality of education.

### 3.4 Analysis of subsistence allowance coverage

**Figure 6:**
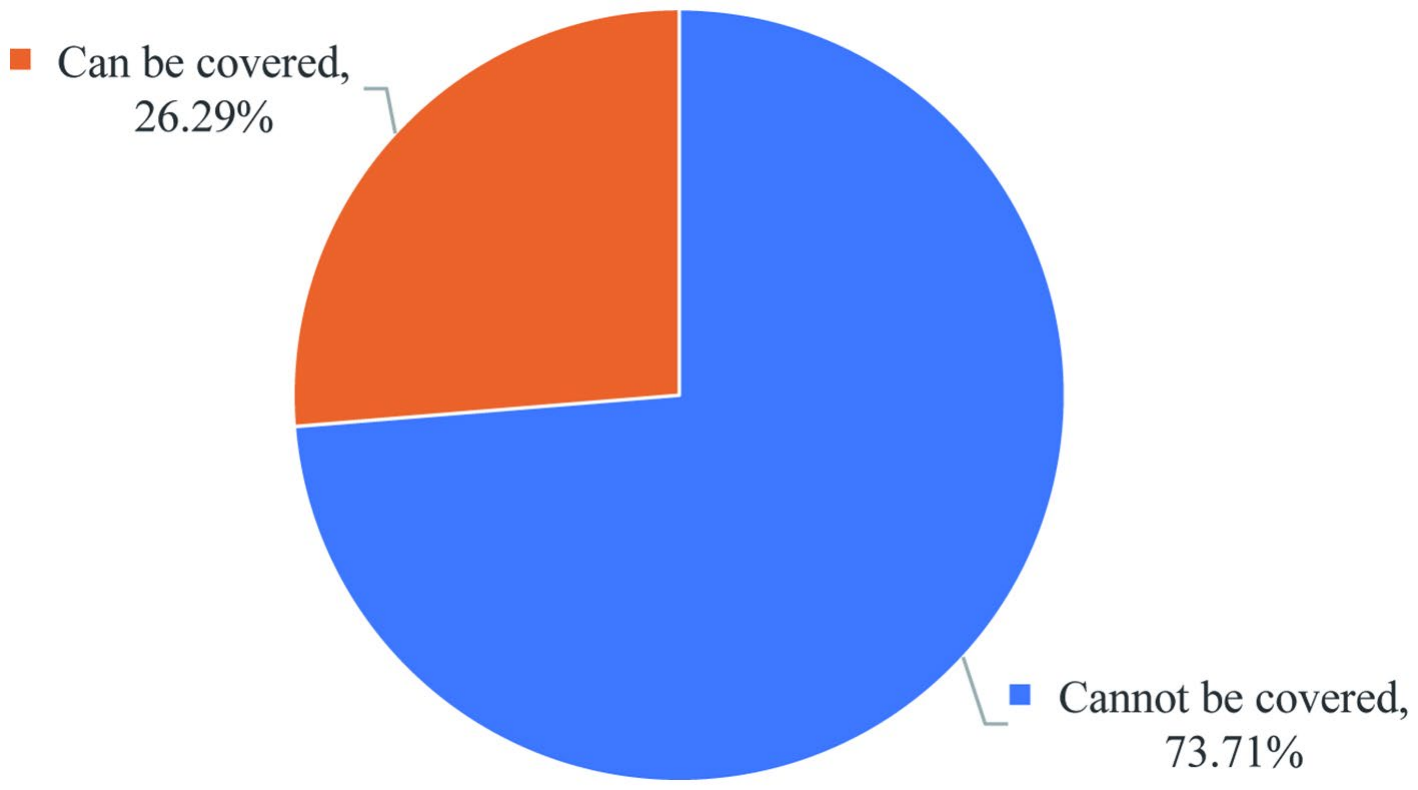
Proportion of graduate students reporting whether their monthly grants/scholarships cover daily expenses.

Figure 6 shows that only 26% of graduate students believe that the monthly subsidy covers their daily expenses (Figure 6). This financial strain aligns with Maslow’s hierarchy of needs theory, which posits that basic physiological and safety requirements must be satisfied before individuals can pursue higher-order cognitive or self-actualization goals like scientific research (11). Given that graduate students—unlike their employed peers—rely solely on institutional subsidies, unmet subsistence needs may explain observed psychological stressors and reduced research engagement. This result suggests that it is necessary for domestic educational institutions to improve the economic treatment of graduate students and ensure their peace of mind in scientific research, which is a necessary condition for stabilizing graduate students and improving scientific research output.

### 3.5 Scientific research funding

**Figure 7:**
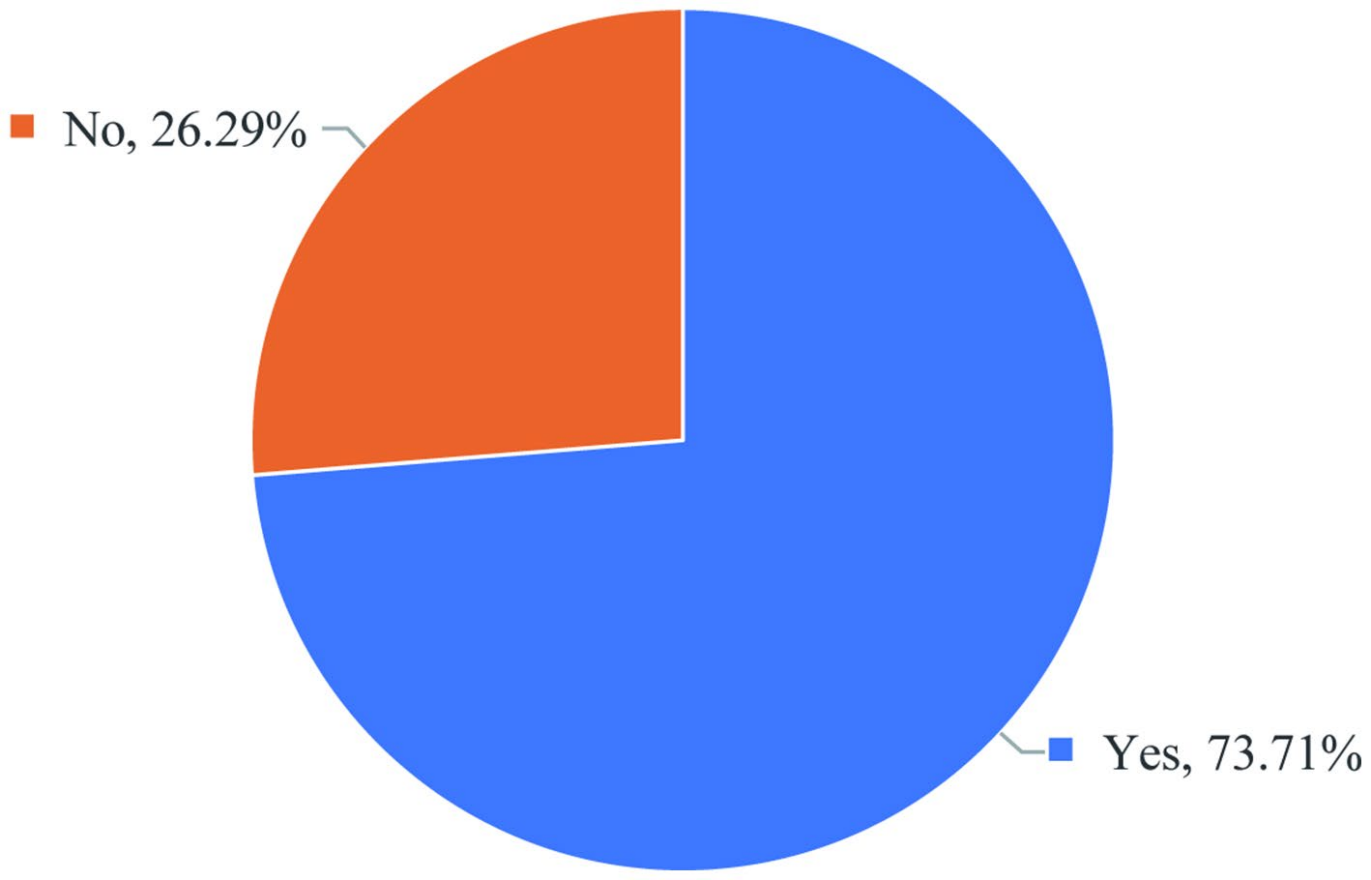
Percentage of graduate students reporting sufficient research funding.

As can be seen from Figure 7 of the survey results, 26% of graduate students still have insufficient research funding (Figure 7). Basic medical research requires a certain amount of money. Basic medicine is supported by experimental data. A large number of experiments need to be done. These experiments need sufficient funding. This data shows that relevant domestic educational institutions need to increase investment in scientific research funds and optimize the allocation mechanism of funds, which is an important material guarantee for promoting basic medical scientific research innovation and improving graduate students ‘scientific research practice ability.

### 3.6 Academic participation and influencing factors

**Figure 8:**
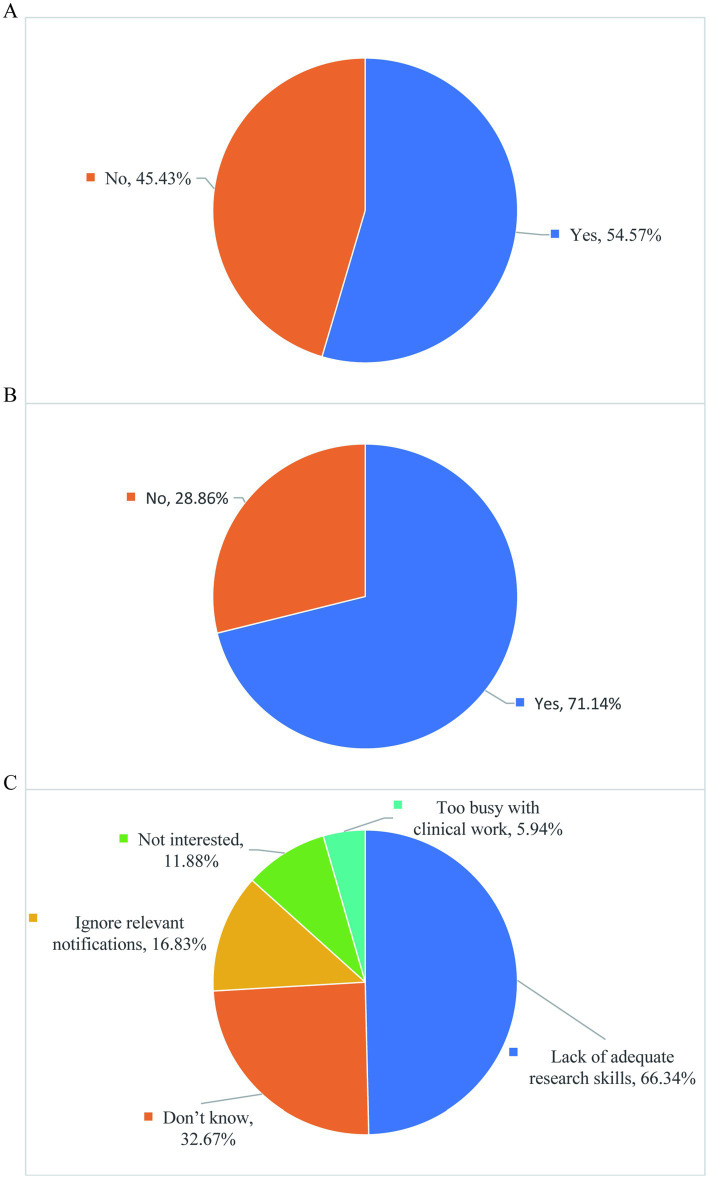
Academic participation and influencing factors. **(A)** During the training period, did your supervisor arrange for you to attend any international or domestic academic conferences. **(B)** Are you actively participating in academic activities. **(C)** Reasons for not participating in academic activities or applying for graduate research topics.

54.7% of graduate students attended international or domestic academic conferences, and there is still room for improvement (Figure 8A). Our survey results show that 71.14% of graduate students actively participate in academic activities, and there are still some students who are not motivated to participate in academic activities (Figure 8B). The reasons for insufficient enthusiasm or failure to apply for graduate subjects mainly include the following aspects: Too busy with clinical work, not interested, ignore relevant notifications, lack of adequate research skills, and do not know (Figure 8C). It is recommended that domestic educational institutions formulate relevant policies to enhance the attraction and participation of academic activities. Such measures would be beneficial in broadening students’ academic vision and enhancing their scientific research competitiveness. These initiatives are closely related to the academic growth of graduate students and the improvement of educational quality.

### 3.7 Current status of scientific research experience before admission

**Figure 9:**
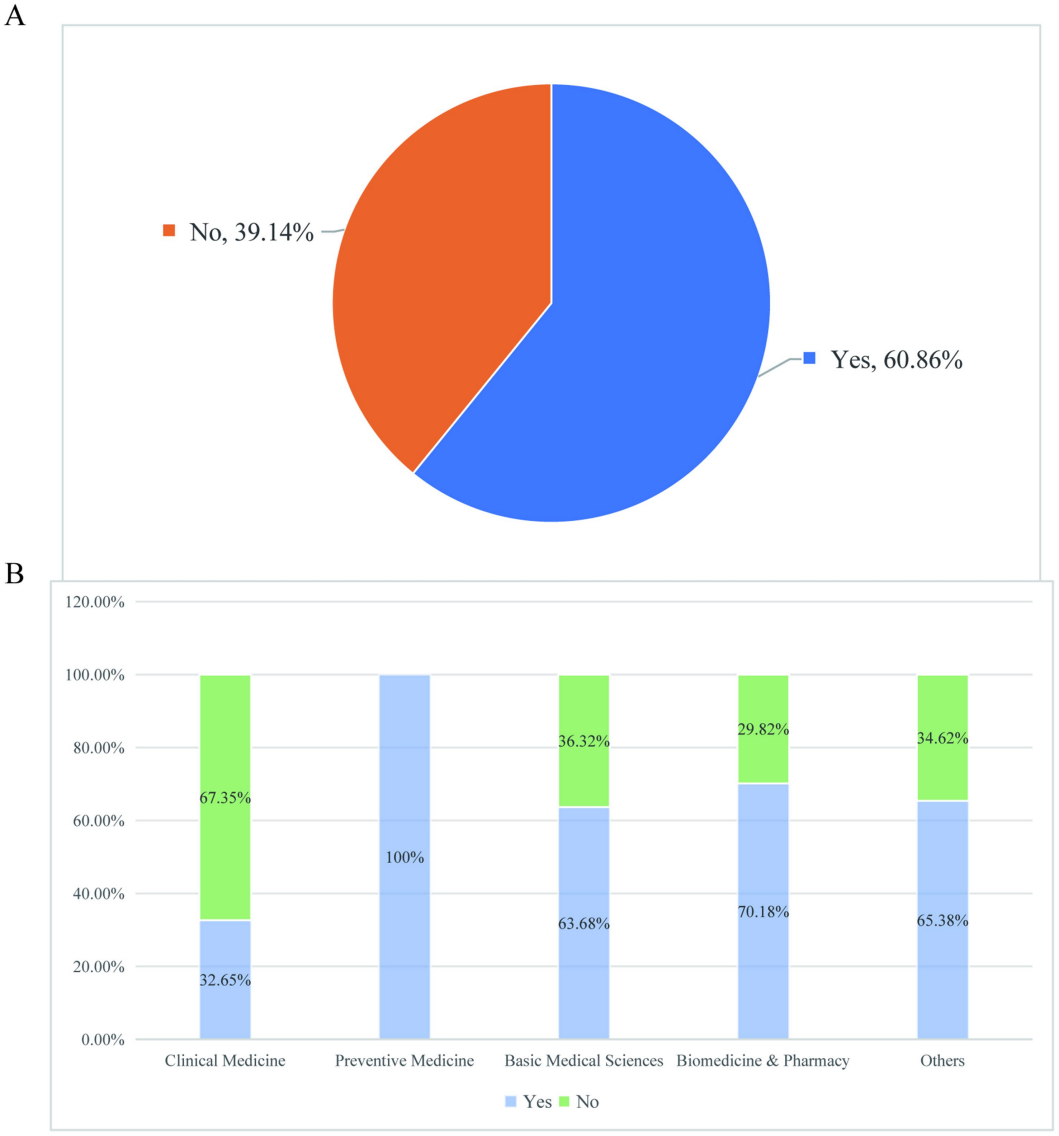
Current status of scientific research experience before admission. **(A)** The proportion of graduate students who have previously been exposed to research work before admission. **(B)** The proportion of graduate students in basic medicine from different undergraduate majors who received research training before admission.

According to the survey results, nearly 1/3 of graduate students have not been exposed to scientific research before entering school, and the proportion of undergraduate graduate students in clinical medicine is higher, and some undergraduate graduate students in other majors have not received scientific research training (Figure 9). Early contact with scientific research, early participation in scientific research projects and integration into scientific research teams are not only conducive to learning scientific research technology in advance, but also conducive to early contact and communication with team members. There are not many opportunities for clinical undergraduates to really contact scientific research, and academic graduate students often start to contact experiments in the second year of graduate students. This survey result provides direction for relevant domestic educational institutions to adjust the connection mechanism between undergraduate and graduate scientific research education, and helps to build a coherent scientific research training system.

### 3.8 Graduate students’ satisfaction with the academic guidance of their supervisors

**Figure 10:**
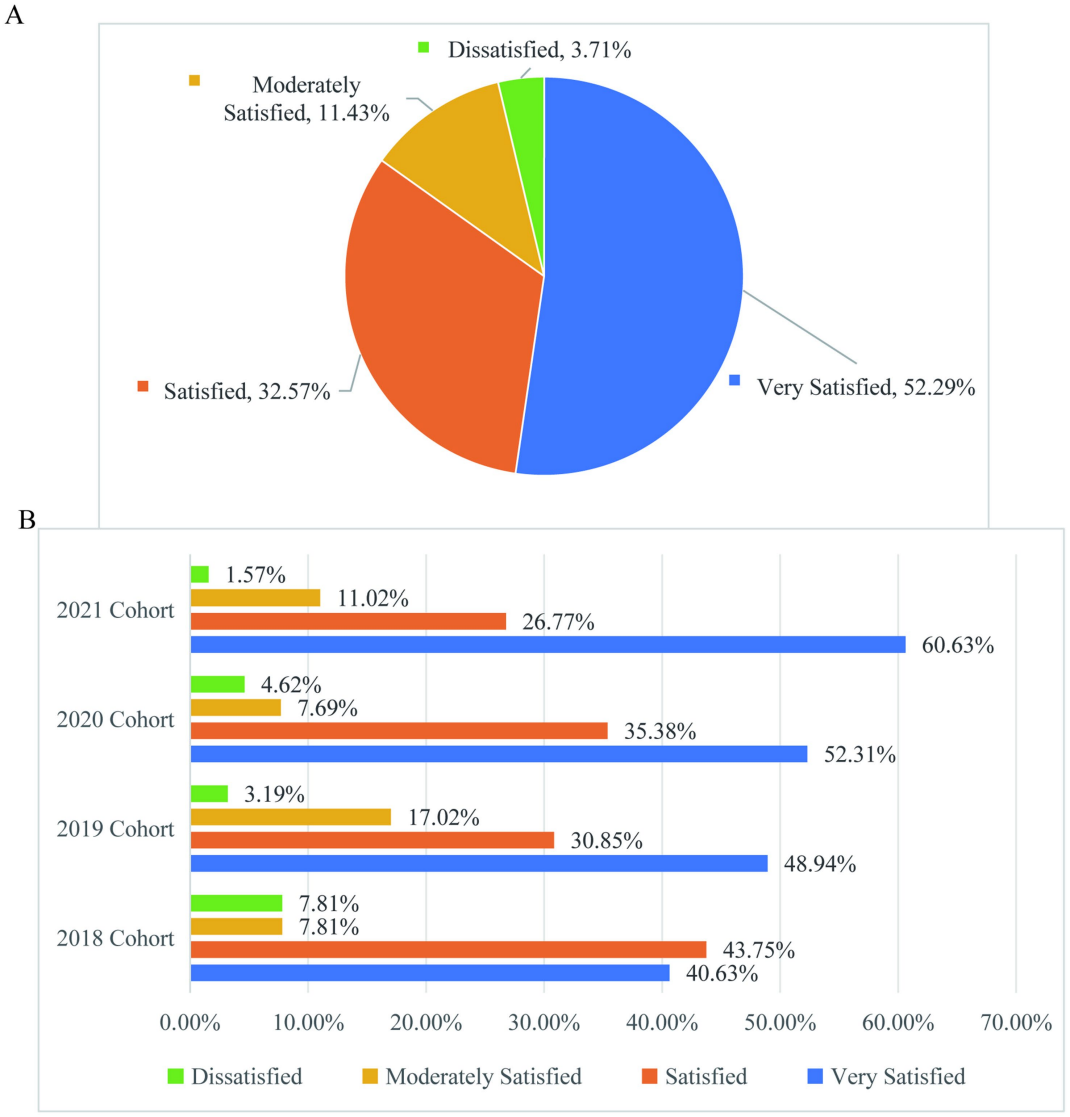
Graduate students’ satisfaction with the academic guidance of their supervisors. **(A)** Distribution of satisfaction levels among graduate students regarding academic guidance from their supervisors. **(B)** Distribution of satisfaction levels among graduate students of different grades regarding their advisors’ research and academic guidance.

Figure 10 of the survey results show that the overall satisfaction of graduate students with the scientific research academic guidance of their supervisors reaches 96.29% (Figure 10A). The proportion of graduate students in different grades who are very satisfied with the scientific research academic guidance of their supervisors exhibits a downward trend with the increase of cohorts (Figure 10B). It is recommended that supervisors pay increased attention to the guidance of senior graduate students. When considering these findings in relation to Figure 8C, it becomes evident that reasons for not participating in research activities include a lack of intrinsic motivation, self-directed learning skills, and possibly time management issues. This suggests that educational institutions at all levels pay attention to the continuous and targeted optimization of supervisor guidance, including fostering intrinsic motivation and self-directed learning skills among graduate students. Establishing a dynamic guidance evaluation mechanism can ensure that graduate students at different stages can obtain high-quality guidance. Additionally, promoting a culture of self-directed learning could help students take more initiative in their research activities, thus improving their satisfaction with academic support from their supervisors.

### 3.9 Graduate student team work atmosphere satisfaction and difference analysis

**Figure 11:**
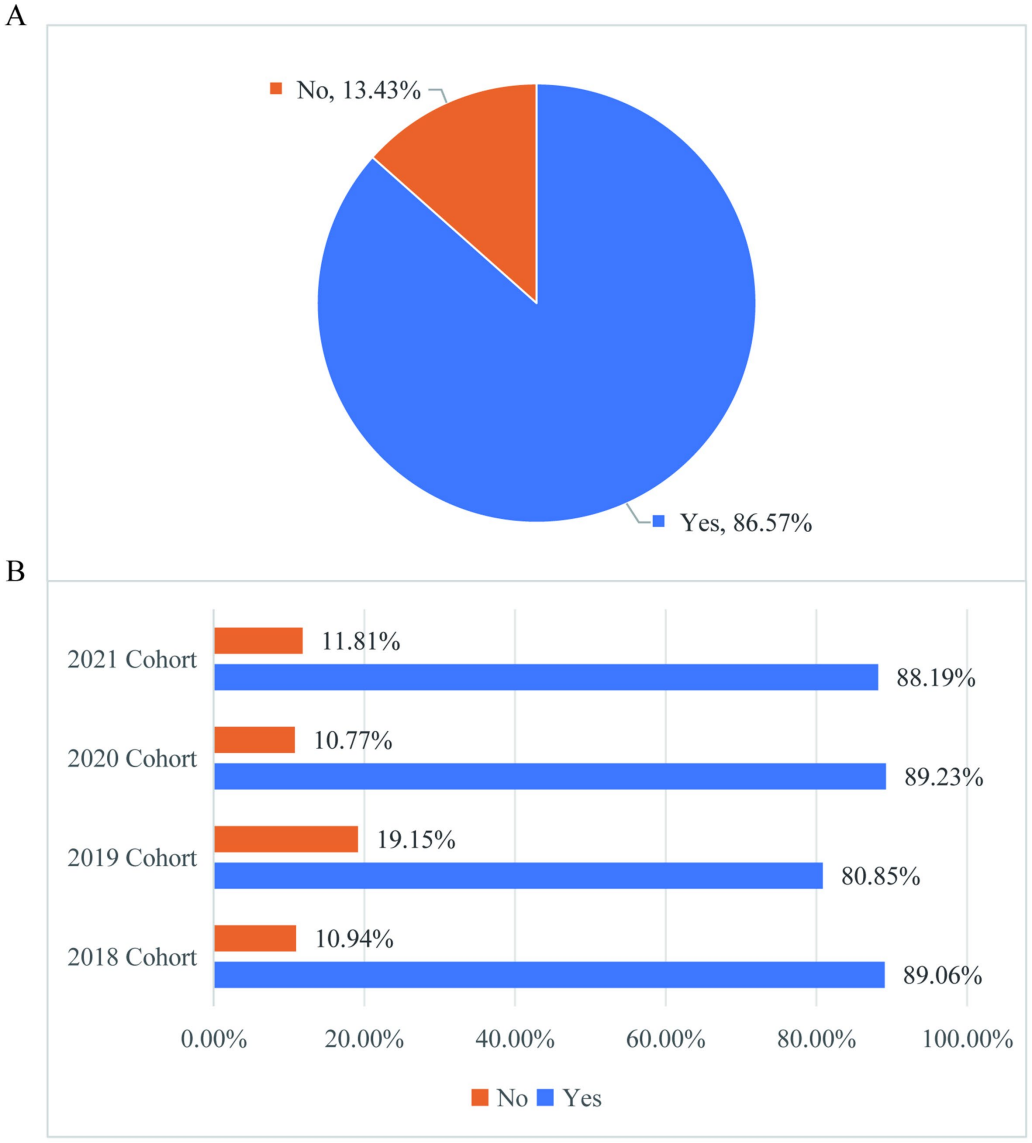
Graduate student team work atmosphere satisfaction and difference analysis. **(A)** Distribution of the proportion of graduate students who like the working atmosphere of their current group. **(B)** The distribution of the proportion of graduate students in different grades who like the work atmosphere of their current group.

Figure 11 of the survey results show that 86.57% of the overall graduate students like the working atmosphere of their group (Figure 11A), and the difference in the proportion of graduate students in different grades like the working atmosphere of their group is reflected in the graduation cohort (2019 cohort) (Figure 11B); greater attention should be paid to students’ life, research, and future career or academic pathways of graduate students in graduation grade. Good team atmosphere is an important factor to improve graduate research efficiency and mental health. This study provides evidence for educational institutions at all levels to optimize team building strategies and pay attention to the special needs of graduate students.

### 3.10 Graduate students’ overall satisfaction with their current graduate life

**Figure 12:**
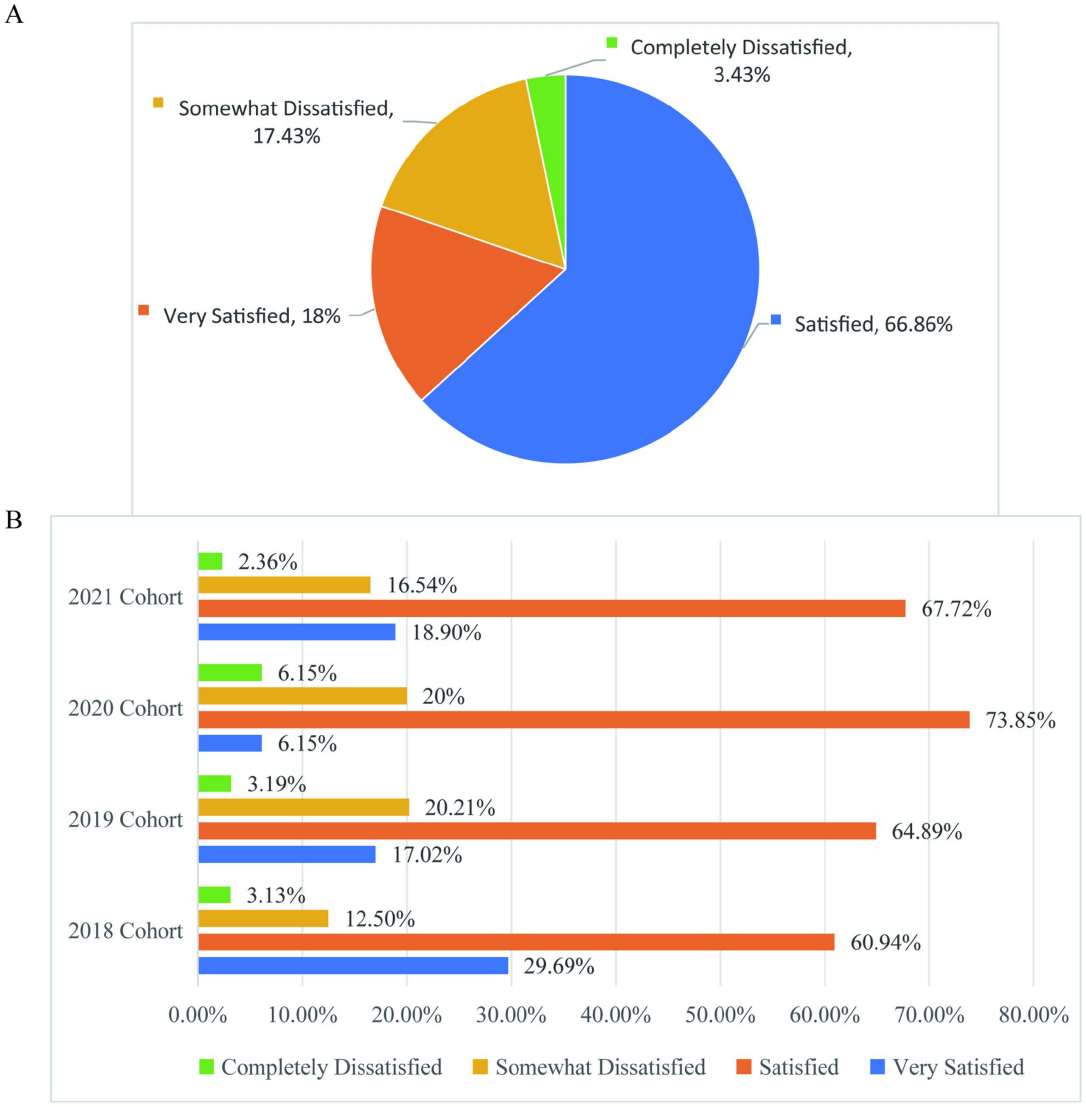
Graduate student’s overall satisfaction with their current graduate life. **(A)** The satisfaction of graduate students with their current graduate student life. **(B)** Satisfaction of graduate students from different grades with their current graduate life.

Figure 12 of the survey results show that the overall dissatisfaction rate of graduate students with the current graduate life is 20.86%, the dissatisfaction rate of graduate students in different grades with the current graduate life is not different by about 20%, and the graduation grade (2018 cohort) has decreased (15.63%) (Figure 12). Therefore, educational institutions at all levels comprehensively improve graduate students’ life satisfaction, involving comprehensive improvement in many aspects of life, study and scientific research, which is the key to creating a high-quality educational ecology and attracting and retaining outstanding talents.

## 4 Recommendations and countermeasures

### 4.1 Enhance graduate student adaptation and academic experience

To enhance their academic experience and adapt more effectively to their new environment, graduate students can implement several proactive strategies. These include embracing SDL, which empowers students to take charge of their educational journey and develop critical thinking skills (12). Additionally, fostering intrinsic motivation can drive students to engage more deeply with their studies and research (13). Seeking academic support early is crucial, as it helps to address challenges promptly and leverage available resources. Engaging in peer learning and networking not only provides a platform for mutual support and knowledge exchange but also enhances students’ social integration and professional development.

### 4.2 Actively guide graduate students to adjust their mentality and adapt to their majors

It is recommended that schools, colleges, and supervisors establish mechanisms to guide graduate students with multi-disciplinary backgrounds to adapt to graduate studies as soon as possible. It is suggested that supervisors actively listen to students, adjust expectations appropriately, and build trust while providing academic guidance (14, 15), and gradually adjust to the transition in graduate school in a positive mood (16).

From undergraduate to graduate students, graduate students need to change their learning thinking mode, actively learn, adjust their mentality, adapt to their majors (17), build a solid psychological and knowledge foundation for subsequent learning and scientific research, and provide demonstrations for relevant educational institutions at all levels to deal with students ‘professional adaptation problems.

### 4.3 Establish multiple communication channels, strengthen information exchange, and timely discover and solve problems

Establish mechanisms at school and college levels to ensure that supervisors have sufficient time to guide graduate students.

Supervisors invest more time and energy in graduate students: In the process of scientific research, timely and effective information exchange is extremely important (18, 19). This kind of communication is two-way, the supervisor needs to keep abreast of the student’s work progress, and the student needs to get corresponding help from the supervisor (20, 21).

Graduate students are proactive: The supervisor is busy with work, so it is encouraged that graduate students think about what problems they need to solve before communicating with the supervisor, and how to solve the problems. When communicating with the supervisor, try to save time and solve the problems efficiently (22). For introverted students who are not good at communication, they should try their best to improve their ability to express themselves, dare to ask questions, not afraid of mistakes, not afraid of criticism, always have an apprentice mentality, consult with supervisors modestly, try to consult face-to-face, and solve problems in writing when necessary; For those students who do not like supervisors and are afraid to communicate with supervisors, it is preferable that they carefully consider whether they understand their supervisors clearly and are really interested in their research direction. If necessary, they can apply to the school for replacement of supervisors.

In medical education, critical thinking and problem-solving skills are essential (23). It is suggested that supervisors pay more attention to guiding students to actively discover problems and actively seek solutions (24).

### 4.4 Reasonable arrangement of time, balance between scientific research and life

Besides scientific research, how to live happily is also a very important thing. Many graduate students often complain about the difficulty of scientific research, which makes it difficult to balance the contradiction between scientific research and life (25), which leads to many graduate students having mental health problems (26, 27). There is a need for graduate students to formulate detailed study and research plans, clarify task priorities, and ensure efficient completion of work.

Learn to set time for rest and relaxation, avoid overwork, actively participate in social activities, expand interpersonal networks, relieve scientific research pressure, and achieve a balance between scientific research and life (28).

### 4.5 Increase funding at multiple levels to solve worries and ease scientific research

The state, society, schools, colleges and supervisors invest more funds through multiple channels to improve the treatment of graduate students and ensure the life and scientific research of graduate students (29). Improve the fund management and supervision mechanism to ensure the rational distribution and use of funds, and provide an example for the construction of fund guarantee mechanism for education-related institutions at all levels.

### 4.6 Build platforms, actively participate in academic activities and broaden their horizons

Increase the enthusiasm of graduate students to participate in academic activities or submit projects (30, 31). Colleges and universities can improve the overall level of scientific research and teaching by building innovation platforms and academic heights and optimizing evaluation and incentive mechanisms (32, 33). At the same time, we will expand academic publicity, improve the content of academic activities, and increase the benefits of applying for projects, so as to improve the initiative of graduate students.

Graduate students are encouraged to actively engage in academic activities and research projects aligned with their professional interests, thereby enhancing their academic competence, career development, and personal growth through increased self-directed initiative (34).

With the strong support of the state for scientific research, the speed of scientific and technological development in China is constantly improving, the level of scientific research is constantly improving, various new scientific research achievements are changing with each passing day, and many graduate textbooks are not updated in time, introduced comprehensively, and the content is not deep. Graduate students’ daily study is different from undergraduate study. During their study, graduate students will have various academic lectures, conferences and opportunities for exchange and study. In addition to completing experimental tasks in the laboratory every day, it is preferable that basic medical graduate students actively participate in professional-related learning and enrich themselves through various channels. Extensive communication can play a positive role in releasing the pressure brought by the subject and solving professional problems during graduate school. At the same time, active contact with the forefront of scientific research is conducive to the cultivation of scientific research thinking, the expansion of knowledge, the improvement of learning and inquiry ability and the cultivation of scientific research interest (35).

### 4.7 Early contact with scientific research and adaptation to scientific research roles

In addition to the study of basic courses, Yanyi makes good use of spare time, reads a lot of relevant literature, actively participates in subject research, and learns experimental techniques in advance, which is of great help to enter the laboratory and participate in scientific research activities quickly in the future (36).

For graduate students who have just come into contact with scientific research, it is also indispensable for supervisors to give more patient and specific guidance, reasonably arrange literature reading tasks, regularly assess learning effects (37), and provide practical paths for the early cultivation of scientific research talents in domestic colleges and universities.

### 4.8 Strengthen graduate student guidance, attach importance to team building, and make efforts to improve graduate student satisfaction by “four-in-one”

Supervisors are the first responsible person for postgraduate training. Supervisors strengthen their own learning (38), attach importance to life care and academic guidance for graduate students; schools and colleges give supervisors training and promotion opportunities (39, 40).

The ideal academic environment is considered to prepare students for future efforts, development, and social well-being (41). Competitive, stressful, threatening, or authoritative environments de-motivate students, while collective, collaborative, and supportive environments are motivating factors (42). It may be useful for supervisors to pay attention to the creation of a good atmosphere within the scientific research team, good interpersonal relations, a united and cooperative team, and an upward scientific research atmosphere (43, 44); schools and colleges should create a wide range of good academic atmosphere.

Actively implement the double supervisor system (45, 46), broaden the vision of graduate students, improve their practical ability, and lay a solid foundation for graduate research and career development (47).

Implement the four-in-one postgraduate training mechanism of “school, college, discipline and supervisor,” pay attention to the whole process of postgraduate “life, psychology, study, scientific research and future development” (48), and provide a model for innovation of postgraduate training mode in educational institutions.

## 5 Discussion

Our study reveals several critical findings regarding the cultivation of top-tier basic medical graduate students under China’s “Basic Discipline Top-Student Training Program 2.0.”

The mentor-mentee relationship emerges as a crucial factor in graduate education (49). Only 30% of students prioritize seeking guidance from their supervisors when encountering difficulties, and satisfaction with mentorship shows a declining trend among senior students. This phenomenon likely reflects a mismatch between supervisors’ expectations of greater research autonomy for advanced students and their actual guidance approaches (50). Notably, despite high overall satisfaction with supervisors’ guidance and regular access to supervisors (80.85% reported meeting at least once a week), a significant number of students still encounter learning difficulties and prefer seeking help from peers. This suggests that while supervisor support is generally available, students may face internal barriers to effective communication. These barriers could include personal characteristics such as introversion, poor communication skills, or fear of communication, rather than issues with the supervisors themselves.

Financial constraints represent another significant challenge. Our data indicates that 26.29% of students struggle with inadequate living stipends, while 26.29% face insufficient research funding—a situation at odds with national policies advocating for improved graduate student support. These financial pressures are particularly detrimental in basic medical research (51), where experimental costs are substantial, and may force students to take on part-time work, compromising their research engagement.

The study also uncovers a concerning cycle of research skill development: 66.34% of students avoid academic activities due to perceived competence gaps, exacerbated by the fact that nearly two-thirds enter graduate programs without prior research experience. While 86.57% of students report positive team environments, satisfaction declines among graduation-year cohorts, highlighting the psychological challenges associated with the long, uncertain research cycles characteristic of basic medical science (52).

These findings collectively reflect implementation gaps in localizing the national “Top-Student Training Program 2.0” policy, particularly in resource allocation and policy execution. As a designated national training base for elite basic medical students, Central South University’s experience offers valuable insights.

Under the background of the national vigorous implementation of “Basic Top-notch 2.0” (53), how to comply with policies, make good use of policies, overcome systemic challenges, and cultivate top-notch Basic scientific research talents who are expected to lead the development of basic medicine in China, and achieve future breakthroughs in scientific frontiers? As important bearers of glorious mission, basic medical graduate students play an important role. It is recommended that the state, society, educational institutions, and supervisors gain a deeper understanding of the life, study, and scientific research experiences of graduate students. It is advisable to target their specific needs and establish comprehensive support mechanisms that encompass various aspects such as daily life, academic pursuits, and research activities (28, 54). Additionally, it would be beneficial to create follow-up channels that align with their further study and development aspirations, ensuring that they receive the necessary assistance and guidance (55). Let the training mode be more perfect, the training mechanism be more perfect, the leading and demonstration role of the training plan for top students in basic disciplines be more prominent, form a training system for top talents in basic medical disciplines with China characteristics and world-class level, cultivate a group of outstanding talents who bravely climb the scientific peak and promote the development of basic medicine in China, and inject continuous innovation power into the vigorous development of medical cause in China.

## Data Availability

The original contributions presented in the study are included in the article/supplementary material, further inquiries can be directed to the corresponding author.
